# LPA-Tuning CLIP: An Improved CLIP-Based Classification Model for Intestinal Polyps

**DOI:** 10.3390/s26061764

**Published:** 2026-03-11

**Authors:** Zumin Wang, Jun Gao, Wenhao Ping, Jing Qin, Changqing Ji

**Affiliations:** 1 College of Information Engineering, Dalian University, Dalian 116622, China; wangzumin@dlu.edu.cn (Z.W.); gyou1t4@gmail.com (W.P.); 2 College of Software Engineering, Dalian University, Dalian 116622, China; qinjing@dlu.edu.cn; 3 College of Physical Science and Technology, Dalian University, Dalian 116622, China

**Keywords:** multimodal endoscopic diagnosis, polyp classification, CLIP fine-tuning, structured prompts

## Abstract

Background and Objective: Accurate classification of intestinal polyps is crucial for preventing colorectal cancer but is hindered by visual similarity among subtypes and endoscopic variability. While deep learning aids in diagnosis, single-modal models face efficiency–accuracy trade-offs and ignore pathological semantics. We propose a multimodal framework that integrates endoscopic images with structured pathological descriptions to bridge this gap. Methods: We propose LPA-Tuning CLIP, which incorporates three key innovations: replacing CLIP’s instance-level contrastive loss with cross-modal projection matching (CMPM) with ID loss to explicitly optimize intraclass compactness and interclass separation through label-aware image-text similarity matrices; introducing structured clinical semantic templates that encode WHO diagnostic criteria into hierarchical text prompts for consistent pathology annotations; and developing medical-aware augmentation that preserves lesion features while reducing domain shifts. Results: The experimental results demonstrate that our proposed method achieves an accuracy of 85.8% and an F1 score of 0.862 on the internal test set, establishing a new state-of-the-art performance for intestinal polyp classification. Conclusions: This study proposes a multimodal polyp classification paradigm that achieves 85.8% accuracy on three-subtype classification via endoscopic image-pathology text joint representation learning, outperforming unimodal baselines by 8.7% and a multimodal baseline by 4.3%.

## 1. Introduction

The incidence of intestinal diseases is increasing annually due to rising stress levels and irregular work patterns. As prevalent intestinal lesions, intestinal polyps can develop into cancer if untreated, representing a major cause of cancer-related deaths worldwide. Studies have shown that most intestinal cancers originate from adenomatous polyps [1], highlighting the critical need for early detection, whereas hyperplastic polyps are generally benign, making their accurate differentiation essential. However, conventional colonoscopy faces substantial limitations. To overcome these challenges, computer-aided diagnostic (CAD) systems have emerged as promising solutions.

Early research on polyp classification heavily relied on traditional machine learning methods, primarily involving manual feature extraction. For instance, researchers explored approaches utilizing directional wavelet-based features [2], Support Vector Machines (SVM) combined with hand-crafted features [3], ensemble learning for histopathological images [4], and various general machine learning techniques for colorectal polyp detection [5]. While these methods improved diagnostic efficiency, they were fundamentally limited by their inability to process high-dimensional endoscopic data and capture complex mucosal patterns.

Subsequently, deep learning solutions have demonstrated superior performance in automated feature learning. These advancements encompass leveraging transfer learning techniques from non-medical domains to mitigate the challenge of limited medical data for polyp detection and classification [6]; integrating online and offline three-dimensional deep learning for robust polyp detection in colonoscopy videos [7]; applying deep learning for Kudo’s classification of colon polyps [8]; and evaluating AI-augmented digital systems for histologic classification [9]. Furthermore, researchers have developed improved deep convolutional neural networks for the multi-classification of polyps [10] and explored few-shot learning strategies for automated classification with limited samples [11].

Despite these significant successes, persistent challenges remain in balancing computational efficiency with diagnostic precision for clinical implementation. This includes broader issues in deep learning’s clinical applications [12], where the effects of complex pathological environments on classification performance are often overlooked [13]. Comprehensive reviews have highlighted the evolution of medical imaging paradigms, emphasizing the pivotal role of knowledge distillation strategies [14] and the transition from simple fusion to general large vision-language models [15]. Building on these advances, recent studies have explored the use of large-scale vision-language models such as contrastive language-image pretraining (CLIP) [16] to address medical imaging tasks [17,18,19].

**Figure 1:**
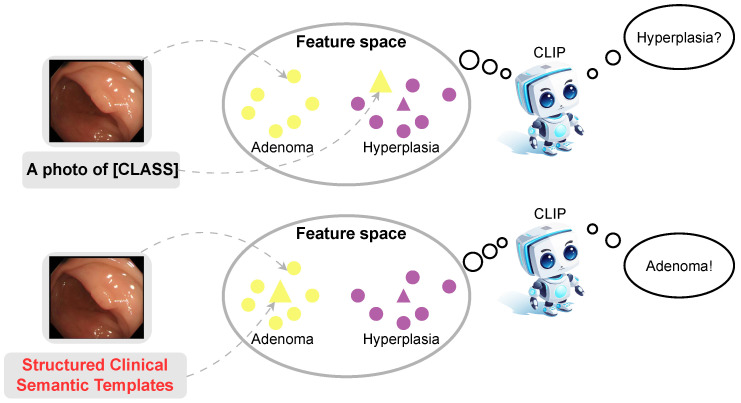
Conceptual comparison of feature space representations between standard prompting and our proposed SCST framework. (**Top Panel**) When using simple class names (e.g., “A photo of [CLASS]”) as text prompts, the resulting semantic anchors (triangles) are vague. This leads to poor class separability in the feature space, where image features (circles) of different categories (e.g., Adenoma vs. Hyperplasia) are mixed, causing model confusion. (**Bottom Panel**) By introducing SCST, the text encoder generates precise, discriminative semantic anchors. This forces the image features to cluster compactly around their respective class centers, significantly increasing the decision margin and resulting in confident, accurate model predictions.

As shown in Figure 1, medical images exhibit intrinsic complexity that cannot be adequately characterized by simple class names alone. While Liu et al. [20] utilized ChatGPT to generate enriched text descriptions, their approach presents three critical limitations for medical applications: lack of standardized pathological terminology, potential hallucination of clinically irrelevant features, and inconsistent description granularity across classes. To overcome these issues, our framework introduces structured clinical semantic template (SCST) with three key improvements: Entity Declaration, Histological Quantification and Clinical Contextualization.

Applying generic CLIP to polyp classification faces a critical ‘semantic gap’. Unlike natural objects, intestinal polyps—specifically the differentiation between Adenoma and Hyperplasia—share high visual similarity, where clinical diagnosis relies on subtle pathological criteria such as surface texture and crypt structure rather than global object shapes. While our SCST framework begins to address the limitations of generic medical prompts, three fundamental challenges persist in fully adapting CLIP for this domain.

First, the substantial domain gap between CLIP’s natural image pretraining and specialized medical imagery hinders the direct transferability of pretrained representations. Second, the highly structured nature of medical texts often leads to the dominance of the textual modality in the feature space; coupled with CLIP’s inherent focus on global image-text matching, this limits the model’s sensitivity to the fine-grained pathological nuances essential for clinical accuracy. Third, the alignment process is further complicated by the requirement for expert-level knowledge, where subjective inconsistencies in manual annotations by different physicians introduce significant noise into the training pipeline.

Motivated by these observations, we propose LPA-Tuning CLIP, a multimodal framework specifically fine-tuned for intestinal polyp classification. The primary contributions of this study are summarized as follows:

Medical-Aware Data Augmentation: To mitigate the domain gap between natural and medical images, we introduce a physics-based augmentation strategy that simulates endoscopic imaging conditions, thereby enhancing the robustness of feature transferability.

Structured Clinical Semantic Template (SCST): To address annotation inconsistency and semantic ambiguity, we design a three-level prompt engineering framework grounded in WHO classification criteria. This standardizes textual descriptions and improves the alignment of fine-grained pathological nuances.

Dual-Loss Optimization Strategy: To alleviate modal asymmetry, we incorporate Cross-Modal Projection Matching (CMPM) with Identity (ID) loss. This synergistic approach simultaneously optimizes intramodal discriminability and intermodal alignment, significantly enhancing classification performance.

## 2. Materials and Methods

### 2.1. Data Collection

The intestinal endoscopy image dataset utilized in this study was collected from 91 patients who underwent intestinal endoscopy. The data collection began in August 2024 at XinHua Hospital, affiliated with Dalian University. The demographic composition of the patient population included 52 females and 39 males, the age range was between 29 and 68, and the average age was 51 years.

**Figure 2:**
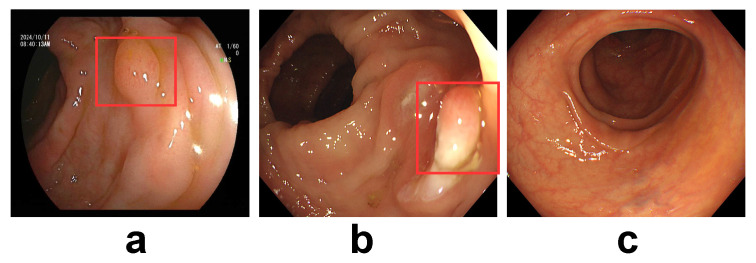
Images of different types of polyps and normal mucosa. The portion of the red bounding boxes indicates the presence of a polyp; (**a**) represents adenoma, (**b**) denotes hyperplasia and (**c**) designates normal mucosa.

This dataset encompasses images of polyps and normal intestinal mucosal tissue for each patient, with all endoscopies being conducted via the Olympus PCF-H290 colonoscopy device (Olympus Corporation, Tokyo, Japan). The dataset comprises a wide array of polyp and normal tissue images, as depicted in Figure 2.

Following a thorough review of the captured polyp images, images that were incompletely captured or lacked clarity were excluded, resulting in the retention of a total of 389 endoscopic images. In the present case series, 154 polyps were identified as adenomatous polyps, 147 were classified as hyperplastic polyps, and 88 were determined to be normal mucosal tissues. The distribution of these data is outlined in Table 1, which accurately reflects the diversity and characteristic distribution of the dataset. All the samples were labelled by three senior clinical experts to ensure the accuracy and consistency of data and to provide high-quality training data for subsequent polyp classification studies.

The ground truth labels were primarily determined by the histopathological reports. To ensure the visual validity of the training samples, a rigorous three-expert consensus protocol was employed. Three senior gastroenterologists independently reviewed the mapping between endoscopic images and pathology records. Any ambiguous cases—where experts disagreed on image quality or lesion correspondence—were subjected to a group discussion. If a consensus could not be reached, the sample was excluded from the dataset to ensure label reliability.

To strictly prevent data leakage and ensure reliable generalization evaluation, the dataset was split at the patient level rather than the image level. Specifically, all endoscopic images belonging to a single patient were assigned exclusively to either the training set or the testing set.

Although the dataset size is constrained by clinical availability, we employed a rigorous medical-aware data augmentation strategy (detailed in Section 2.2.2) to artificially expand the training diversity. This strategy simulates various endoscopic conditions, effectively mitigating the overfitting risk associated with limited sample sizes.

To increase the precision of polyp classification, this study furnished supplementary textual descriptive information, namely the synopsis of the relevant enteroscopic pathology report for each sample. This segment of the text was provided by expert physicians from Xinhua Hospital affiliated with Dalian University, serving as pivotal auxiliary information to the dataset, thereby augmenting the discriminative capacity of the model.

### 2.2. The Proposed LPA-Tuning CLIP

**Figure 3:**
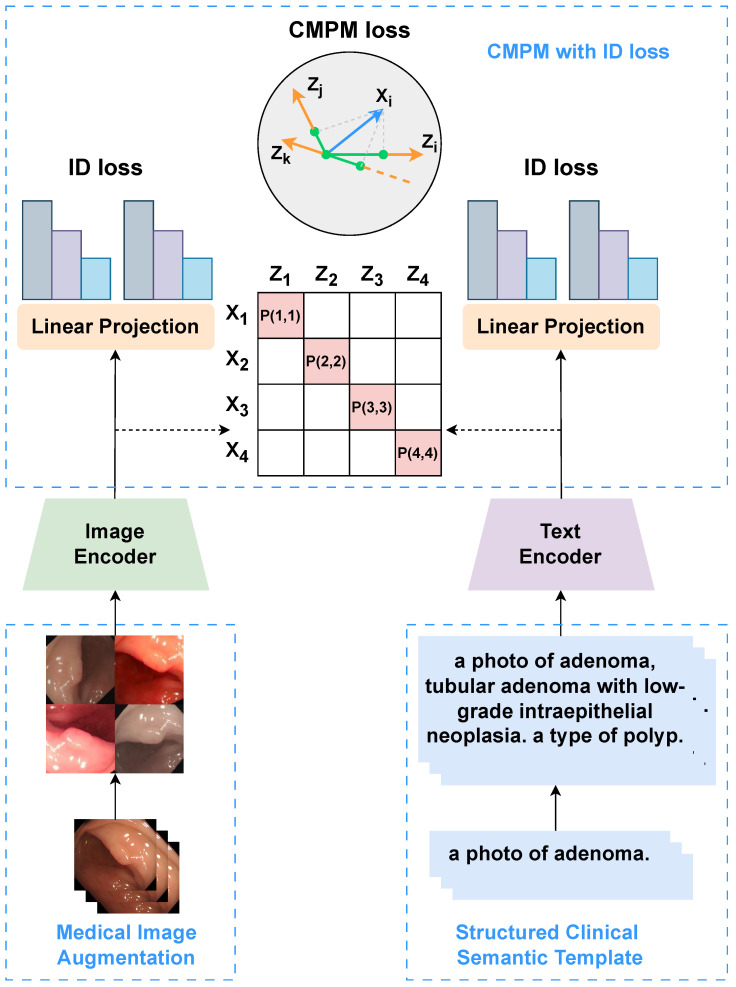
The overall architecture of the proposed LPA-CLIP framework. The pipeline processes inputs in three stages: (1) Input Construction (**Bottom**): Endoscopic images undergo Medical Image Augmentation to simulate clinical variations, while text inputs are enriched using our SCST to provide fine-grained supervision. (2) Feature Encoding (**Middle**): The augmented images and structured prompts are processed by the Image Encoder and Text Encoder, respectively, to extract high-dimensional representations. (3) Optimization Objectives (**Top**): The model is optimized using a joint loss function: CMPM Loss aligns the global distributions of visual and textual features (visualized by the matching matrix and hypersphere), while ID Loss enforces class separability via linear projections. This dual-constraint mechanism ensures robust domain adaptation for polyp classification.

Our work builds upon the contrastive language-image pretraining (CLIP) framework, which learns visual concepts through natural language supervision. As detailed in Section 2.2.1, the CLIP dual-encoder architecture aligns image and text embeddings in a shared latent space. To adapt this powerful foundation for medical image analysis, particularly intestinal polyp recognition, as illustrated in Figure 3, we propose LPA-Tuning CLIP with three key innovations: (1) medical image augmentation (Section 2.2.2) preserves pathological features while diversifying training data; (2) medical prompt tuning (Section 2.2.3) optimizing text embeddings via structured clinical descriptions; and (3) cross-modal projection matching with ID Loss (Section 2.2.4) enhances both feature alignment and class discrimination. The synergistic integration of these components enables robust domain adaptation for medical applications.

#### 2.2.1. Contrastive Language-Image PreTraining (CLIP)

The CLIP model [16] is a cross-modal contrast learning framework that is based on large-scale image-text pairs, which realizes the fine-grained alignment of the semantic space via a bimodal joint embedding mechanism. The model’s primary input resides in its dual-branch coding architecture and contrast pretraining paradigm. The visual branch employs ResNet [21] or Vision Transformer [22] to extract image features, whereas the textual branch generates linguistic representations via a transformer. The learning objective is formulated as:

(1) $$L_{CLIP}=-\frac{1}{2N}\sum_{i=1}^{N}\left[log\frac{e^{\langle I_{i},T_{J}\rangle /\tau }}{\sum_{j=1}^{N}e^{\langle I_{i},T_{j}\rangle /\tau }}+log\frac{e^{\langle T_{j},I_{i}\rangle /\tau }}{\sum_{j=1}^{N}e^{\langle T_{j},I_{i}\rangle /\tau }}\right]$$

where τ is a temperature hyperparameter, and 〈·,·〉 denotes cosine similarity.

The image feature vector $I_{i}$ (output from the visual branch) and the text feature vector $T_{j}$ (output from the text branch) are then normalized and projected to a shared latent space $I,T\in R^{N\times D}$, where $I\in R^{N\times D}$ denotes an embedding matrix for *N* endoscopic images, $T\in R^{N\times D}$ denotes an embedding matrix for *N* pathology description text, *i* and *j* denote sample indices in a batch, and *D* represents the shared embedding dimension. The model optimizes the bidirectional contrast loss function by constructing $N^{2}$ graphic and text pairs of samples, maximizing the cosine similarity of positive sample pairs while suppressing the negative sample correlation.

This joint objective ensures mutual alignment between the image and text modalities, thereby enhancing generalization and effectively mitigating the domain bias common in traditional multimodal models.

#### 2.2.2. Medical Image Augmentation

This study employs a composite data augmentation framework, integrating morphological augmentation and photometric augmentation, to address the challenges posed by viewpoint diversity and illumination interference in the context of medical image analysis. It focuses on the physical imaging properties of endoscopic images and clinical application scenarios.

The endoscopic probe rotation operation was simulated, and the central region of the image after constrained rotation retained the complete pathological structure and avoided the loss of key features. The translation and tilt motions of the probe were modelled via a 2D affine transformation matrix A, which is defined as:

(2) $$A=\left[\begin{array}{ccc} scos\;\theta & -ssin\;\theta & t_{x}\\ ssin\;\theta & scos\;\theta & t_{y}\\ 0 & 0 & 1 \end{array}\right],$$

where *s*∼u(0.9,1.1) is a scaling factor sampled uniformly between 0.9 and 1.1, $t_{x},t_{y}$∼u(−0.1w,0.1w) are translational displacements along the x- and y-axes, bounded by ±10% of the image width *w*, and θ∼$u(-15^{\circ },15^{\circ })$ is the tilt angle, restricted to ±15° to ensure realistic motion simulation.

Channel-separated color dithering is employed to independently adjust the intensity of each RGB channel, simulating variations in the light source color temperature. The augmented image intensity $I_{aug}^{c}\left(x,y\right)$ is computed as:

(3) $$I_{aug}^{c}\left(x,y\right)=\beta_{c}\cdot I^{c}\left(x,y\right)+y_{c},c\in \left\{R,G,B\right\},$$

where $I_{c}\left(x,y\right)$ denotes the original intensity at pixel (x,y) in channel *c*, $\beta_{c}$∼u(0.9,1.1) is a luminance factor scaling the intensity, sampled uniformly between 0.9 and 1.1, $y_{c}$∼u(−0.1,0.1) is a brightness bias introducing additive noise, and the resulting pixel values $I_{aug}^{c}\left(x,y\right)$ are clipped to [0, 1] to maintain valid RGB ranges.

This solution achieves a balance between enhanced efficiency and diagnostic validity in endoscopic image analysis through a physically-inspired parametric design, providing a practical solution for resource-constrained clinical deployment scenarios.

#### 2.2.3. Medical Prompt Tuning

**Figure 4:**
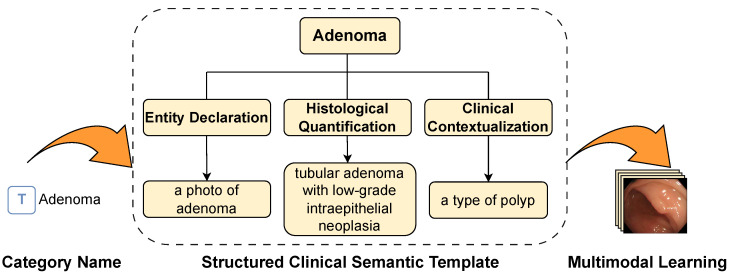
Workflow of SCST for adenoma characterization. Illustrates the three-level semantic hierarchy of SCST applied to adenoma diagnosis: Entity Declaration, Histological Quantification and Clinical Contextualization.

This study proposes a structured clinical semantic template (SCST), as shown in Figure 4, that aims to address the problem of textual semantic ambiguity in medical multimodal tasks. The template generation process is defined as

(4) P(y)=E(y)⊕Q(y)⊕C(y),

where *y* denotes the pathological label and ⊕ represents clinical terminology concatenation. Specifically:

Entity Declaration (E(y))—establishes the global semantic anchor. It standardizes the classification of lesions by explicitly attributing them to major pathological categories (e.g., adenoma vs. hyperplasia). Crucially, this component mitigates the high inter-class visual similarity by enforcing a clear categorical separation at the root level of the text embedding, preventing the model from confusing distinct lesion types that share similar global shapes.

Histological Quantification (Q(y))—the core innovation for fine-grained discrimination. It encodes specific WHO diagnostic criteria into the prompt. By integrating these objective pathological indicators (aligned with the Vienna grading system), Q(y) explicitly guides the visual encoder to attend to diagnostically significant micro-features rather than irrelevant background noise, directly addressing the challenge of subtle texture differentiation.

Clinical Contextualization (C(y))—incorporates the biological behavior and risk level of the lesion. This component associates the lesion with its broader clinical framework, reinforcing the model’s understanding of the diagnostic scenario and ensuring that the learned features align not just with visual patterns, but with clinical management priorities.

As shown in Table 2, by grounding prompts in World Health Organization (WHO) diagnostic criteria, the SCST framework enriches textual representations with high-density clinical knowledge, effectively reducing semantic entanglement and improving fine-grained differentiation without additional computational overhead.

This paradigm significantly improves the accuracy of multimodal semantic alignment by structurally embedding clinical pathology characterization into prompt templates. The template’s information density satisfies:

(5) H(P(y))≤H(E(y))+H(Q(y))+H(C(y)),

where H(.) measures prompt space entropy. It eliminates the need for complex dynamic parameter tuning, emphasizing instead the design of high information-density templates to enhance model performance without incurring additional computational overhead.

#### 2.2.4. Cross-Modal Projection Matching with ID Loss

Although the original CLIP model employs a symmetric bidirectional contrastive loss (InfoNCE) to align image and text features, its objective is strictly instance-level, treating two images of the same polyp class as negative pairs if they come from different samples. This formulation is suboptimal for medical classification, where class-level compactness is essential.

To address this, we adapt Cross-Modal Projection Matching (CMPM) loss [23] into our CLIP fine-tuning framework. Unlike its original application in Person Re-Identification, we repurpose CMPM to align endoscopic visual features with our Structured Clinical Semantic Templates (SCST). By minimizing the KL-divergence between the projection compatibility distributions and the normalized ground-truth labels, CMPM enforces that all visual instances of a specific polyp subtype (e.g., Adenoma) cluster around their corresponding pathological semantic description, rather than just matching single image-text pairs. The CMPM loss is defined as:

(6) $$L_{CMPM}=\frac{1}{2}\left(L_{I2T}+L_{T2I}\right),$$

(7) $$L_{I2T}=-\frac{1}{N}\sum_{n=1}^{N}log\frac{exp(v_{i}^{T}t_{i}/\tau )}{\sum_{j=1}^{N}exp\left(v_{i}^{T}t_{j}/\tau \right)},$$

(8) $$L_{T2I}=-\frac{1}{N}\sum_{n=1}^{N}log\frac{exp(t_{i}^{T}v_{i}/\tau )}{\sum_{j=1}^{N}exp\left(t_{i}^{T}v_{j}/\tau \right)},$$

where $L_{I2T}$ and $L_{T2I}$ denote the image-to-text and text-to-image projection-matching losses, and τ denotes the temperature hyperparameter.

In parallel, to enhance class discriminability in the visual space, we introduce a linear classification head after the CLIP visual encoder and optimize it via cross-entropy loss:

(9) $$L_{ID}=-\sum_{i=1}^{N}\sum_{c=1}^{C}y_{i,c}\log\left(\frac{\exp(w_{c}^{⊤}v_{i}+b_{c})}{\sum_{k=1}^{C}\exp\left(w_{k}^{⊤}v_{i}+b_{k}\right)}\right),$$

where $v_{i}$ is the image feature, $W\in R^{d\times C}$ is the classification weight matrix, and $b_{c}$ is the bias for class *c*.

The total training objective is:

(10) $$L_{Total}=L_{CMPM}+L_{ID}.$$

This combined loss structure promotes both intermodal alignment and intramodal discrimination. While CMPM facilitates semantically structured feature alignment across modalities, ID loss encourages compact intraclass representation and distinct class boundaries. Together, they offer a more robust and fine-grained learning framework for medical image–text understanding tasks.

In summary, this chapter presents a refined CLIP-based framework that incorporates CMPM loss and ID supervision, tailored to address the unique challenges in medical cross-modal alignment. These improvements lay a solid foundation for the subsequent experimental validation. The next chapter will empirically evaluate the proposed method on real-world polyp datasets to demonstrate its effectiveness.

## 3. Experimental Setup

In this study, we established a comprehensive experimental framework to rigorously assess the effectiveness of the proposed LPA-Tuning CLIP method (as described in Section 2.2) for intestinal polyp classification. The evaluation protocol employs standard metrics (accuracy, recall, and F1 score) to compare our fine-tuned CLIP against baselines through three ablation studies.

### 3.1. Compared Configurations

To analyse the contributions of different components of our proposed method, we compared the following model configurations:

Full LPA-Tuning CLIP: The complete model as described in Section 2.2 uses fine-tuned CLIP with medical image augmentation, CMPM with ID Loss, and medical prompt tuning on the basis of pathological text.

LPA-Tuning CLIP without CMPM with ID Loss: Start with the full LPA-Tuning CLIP, but remove the CMPM with ID Loss component. It still uses the fine-tuned CLIP encoders, medical image augmentation, and medical prompt tuning with pathologic text. The image and text features might be combined differently.

LPA-Tuning CLIP without Medical Prompt Tuning: Start with the full LPA-Tuning CLIP, but replace the medical prompt tuning with a simpler text representation. This could be using fixed, generic prompts (images of the [polyp type]) or just the raw class names as the text input. Pair with the images and use the CMPM with ID Loss. It still uses the fine-tuned CLIP encoders and medical image augmentation.

### 3.2. Training Details

All experiments were conducted using the Ubuntu 22.04 operating system. The hardware core utilized two NVIDIA GeForce RTX 4060Ti (16 GB) graphics cards to accelerate model training through parallel computing. The driver version was 535.261.03, and the CUDA version was 12.2. The software development environment employed the PyTorch 2.2.2 deep learning framework, supplemented by the Python 3.11 environment for script writing. Regarding the fine-tuning strategy, the image encoder was initialized with CLIP’s pre-trained weights (VIT/L-14), and we opted for a partial fine-tuning approach by only updating the deeper layers. The text encoder was kept frozen to preserve the semantic knowledge learned from large-scale pre-training.

We utilized the AdamW optimizer, an optimization algorithm that is well suited for training large transformer models, configured with $\beta_{1}=0.9$, $\beta_{2}=0.98$, and $\epsilon =1\times 10^{-8}$. The initial learning rate, which controls the step size during parameter updates, was set to $1\times 10^{-5}$. Weight decay was applied with a value of 0.0005 as a regularization technique to mitigate overfitting. A learning rate scheduler was employed to adjust the learning rate dynamically during training, aiming for better convergence. It begins with a linear warm-up phase over the first five iterations to stabilize training initially, followed by a step decay schedule that reduces the learning rate by a factor of 0.5 every 1000 iterations. The models were trained for a total of 60 epochs using a batch size of 8. The input images were resized to 224×224 pixels to match the input requirements of the pretrained model. The temperature parameter τ used in the contrastive loss calculation was set to 0.02; this parameter scales the similarity scores and influences the separation between positive and negative pairs.

To further reduce the risk of overfitting and evaluate model stability on the small dataset, we leveraged the powerful pre-trained representations of CLIP and implemented a five-fold cross-validation scheme. The dataset was partitioned into five mutually exclusive folds based on patient IDs. The final performance metrics reported are the mean values along with standard deviations, ensuring the statistical significance of the results.

### 3.3. Evaluation Metrics

To quantitatively evaluate the performance of the proposed LPA-Tuning CLIP model in intestinal polyp classification, we employ four standard evaluation metrics: Accuracy, Precision, Recall, and F1-score. These metrics are defined based on the components of the confusion matrix, including True Positives (TP), True Negatives (TN), False Positives (FP), and False Negatives (FN).

Accuracy represents the proportion of correctly predicted samples among the total number of samples:

(11) $$\mathrm{Accuracy}=\frac{TP+TN}{TP+TN+FP+FN}$$

Precision (Positive Predictive Value) measures the accuracy of positive predictions, which is critical in clinical settings to minimize over-diagnosis:

(12) $$\mathrm{Precision}=\frac{TP}{TP+FP}$$

Recall (Sensitivity) indicates the ability of the model to identify all relevant positive samples, which is essential for preventing the omission of potentially cancerous polyps:

(13) $$\mathrm{Recall}=\frac{TP}{TP+FN}$$

The F1-score is the harmonic mean of Precision and Recall, providing a balanced assessment of the model’s performance, especially when dealing with subtle class imbalances:

(14) $$\text{F1-score}=2\times \frac{\mathrm{Precision}\times \mathrm{Recall}}{\mathrm{Precision}+\mathrm{Recall}}$$

For our three-subtype classification task (Adenoma, Hyperplasia, and Normal), these metrics are calculated for each class, and the average results are reported to ensure an unbiased evaluation of the model’s overall diagnostic capability.

## 4. Results

This chapter systematically validates the proposed LPA-Tuning CLIP through (1) performance comparison with state-of-the-art models and (2) ablation studies on critical technical innovation. Our method achieves 85.8% classification accuracy on intestinal polyp datasets, surpassing existing medical multimodal models.

We experiment with different pretraining weights based on CLIP for different visual coder layers and find that the Vision transformer layer performs better as a visual coder than ResNet does, with ViT-L/14 working the best; the performance comparison is shown in Table 3.

### 4.1. Comparison with State-of-the-Art Methods

To rigorously evaluate LPA-Tuning CLIP, we conducted comparative experiments with eight state-of-the-art models spanning two categories: medical-specialized multimodal models pretrained on large-scale medical image-text pairs from diverse modalities (MedCLIP [24], ConVIRT [25], and PMC-CLIP [26]) and general vision backbones pretrained on ImageNet-21k (vision transformer [22], ResNet-152 [21], and EfficientNet-B7 [27]). Furthermore, we extended our benchmark to include recent foundation models like BiomedCLIP [28] and MedMamba [29]. Even against these advanced baselines, our method maintains a competitive edge, largely because the SCST explicitly injects fine-grained pathological logic to guide the classification of visually ambiguous polyps. All the comparative models were trained and evaluated under identical data splits and hyperparameters to ensure fairness. To further guarantee the validity of the evaluation, all baseline models were implemented using their officially released weights and the optimal prompt templates suggested by their original authors. This approach ensures that we evaluate the maximum potential of each method in its intended configuration. As shown in Table 4, LPA-Tuning CLIP demonstrates consistent improvements over both medical multimodal and general vision models.

Compared with medical-specialized multimodal models, our approach achieves a 4.3–6.9% accuracy gain by addressing three key limitations: enhanced domain specificity through endoscopic-focused augmentation, which reduces feature distribution shifts; a structured clinical semantic template using WHO-aligned templates, which improves pathological feature alignment compared to free-text reports; and balanced modality learning where CMPM with ID Loss achieves a text-to-image attention ratio versus the imbalance in the baseline models.

Compared with conventional vision networks pretrained on natural images, our framework achieves 8.7–10.3% accuracy gain through three synergistic mechanisms: cross-modal clinical integration that encodes clinical pathology texts inaccessible to unimodal systems, pathology-specific discrimination reducing adenomatous-hyperplastic polyp confusion via hierarchical feature learning, and endoscopic robustness maintaining stable performance under challenging conditions.

**Figure 5:**
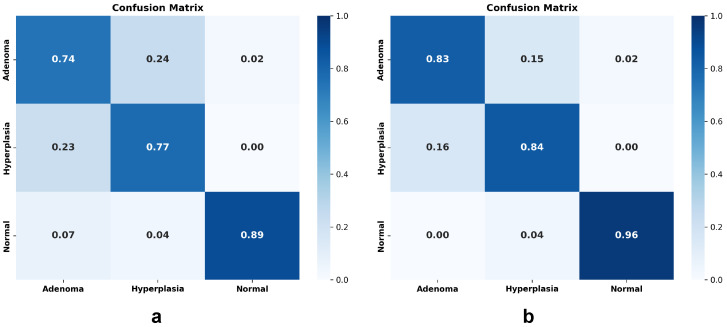
(**a**) Represents the confusion matrix of the original CLIP model on intestinal polyp classification, and (**b**) represents the confusion matrix of LPA-Tuning CLIP, where darker diagonal concentration confirms improved feature separability.

As shown in Figure 5, the confusion matrix reveals that the accuracy improvement is driven by topological transformation of the embedding space to align with pathological diagnostic pathways. As shown in Figure 5b, the confusion matrix not only highlights high sensitivity but also demonstrates robust specificity. The model effectively suppresses False Positives, with only a small fraction of Hyperplastic polyps being misclassified as Adenomas, thereby minimizing the risk of unnecessary interventions.

### 4.2. Critical Performance Factors

To systematically evaluate the contribution of each core component in the LPA-Tuning CLIP framework, we conducted a comprehensive ablation study. Three degraded variants were derived from the full model:

Is the “Medical-Aware Augmentation” step important? As shown in Table 5, ablation studies demonstrate that removing medical-aware augmentation leads to a 2.2% decrease in accuracy (from 85.8% to 83.6%) and a 2.3% reduction in the F1 score on the internal test set. This performance degradation highlights the critical role of augmentation in enhancing model robustness against real-world endoscopic variations, such as lighting fluctuations, motion blur, and mucosal reflections. The results confirm that domain-specific augmentation is indispensable for bridging the simulation-to-reality gap in endoscopic AI.

Is the “Structured Clinical Semantic Template (SCST)” step important? As shown in Table 6, ablation experiments reveal that replacing the SCST with generic class labels leads to a 3.1% accuracy decline (from 85.8% to 82.7%) and a 3.3% lower F1 score. This degradation stems from the loss of WHO-aligned diagnostic hierarchies in text embeddings–generic prompts fail to preserve critical pathological distinctions, causing feature space entanglement. The results prove that the SCST is vital for encoding domain-specific clinical semantics.

Is the “CMPM with ID Loss” step important? As shown in Table 7, disabling the CMPM with ID Loss module resulted in the most significant performance degradation among all ablated components, with a 3.7% accuracy decrease (from 85.8% to 82.1%) and a 3.9% F1 score reduction on the internal test set. This substantial performance decline occurs because CMPM and ID loss provide complementary constraints in the embedding space. Specifically, CMPM ensures the visual encoder captures the precise clinical semantics defined in our SCST. Simultaneously, ID loss prevents visually similar but pathologically distinct categories—such as adenomas and hyperplastic polyps—from overlapping in the feature space. The synergy between these two losses creates a “tighten-and-separate” effect: tightening intra-class variance while maximizing inter-class margins. Without this dual-constraint mechanism, pathological concepts become decoupled from visual features, leading to the observed performance drop. These results underscore that the synergy of CMPM and ID loss is essential for preserving high-precision cross-modal alignment in fine-grained medical tasks.

**Figure 6:**
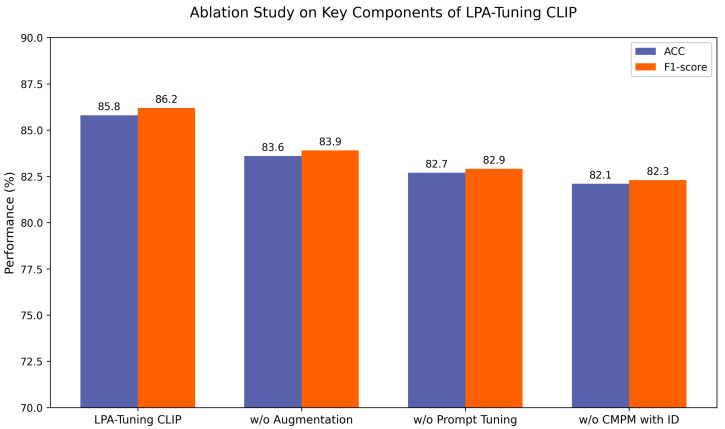
Ablation study on key components of the LPA-Tuning CLIP framework, showing performance degradation (Accuracy and F1-score) when progressively removing critical modules.

Each variant was trained under identical hyperparameter settings and evaluated on the internal test set. As shown in Figure 6, the performance degradation across accuracy and F1 score metrics reveals the criticality of these components, with CMPM with ID Loss exhibiting the most pronounced influence on classification efficacy.

**Figure 7:**
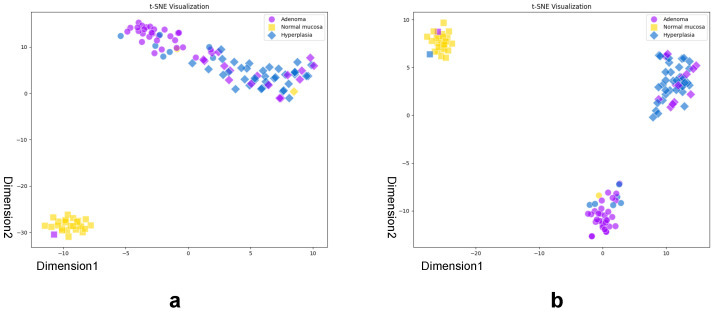
t-SNE visualization of LPA-Tuning CLIP and w/o CMPM with ID. (**a**) shows LPA-Tuning CLIP t-SNE and (**b**) shows w/o CMPM with ID t-SNE. Points are colored by ground-truth labels: adenoma (purple), hyperplasia (blue), and normal mucosa (yellow). Marker shapes indicate predicted labels: adenoma (circle), hyperplasia (rhombus), and normal mucosa (square). Visualization parameters: marker size = 150, alpha = 0.6.

To further validate these findings at the feature level, Figure 7 presents t-SNE visualizations of the multimodal embedding spaces for the CMPM with ID Loss ablated variant. The full model (Figure 7a) shows well-separated clusters for normal mucosa (yellow) and polyps (blue and purple), with text-image pairs (matching shapes) tightly colocated, demonstrating effective cross-modal alignment. Ablating CMPM with ID Loss (Figure 7b) causes pathological concept drift, where hyperplastic polyps incorrectly overlap with adenomatous polyps. These observations quantitatively corroborate the macrolevel performance decreases reported in Figure 6.

## 5. Discussion

Our study introduces LPA-Tuning CLIP, a novel multimodal framework that synergizes endoscopic imaging with structured pathological descriptions to improve intestinal polyp classification. The model achieved 85.8% accuracy on internal testing, outperforming unimodal image-based models by 8.7% and baseline CLIP by 4.3%. This demonstrates its efficacy in distinguishing visually similar polyp subtypes under complex endoscopic conditions—a critical advancement for colorectal cancer prevention.

### 5.1. Synergistic Analysis of the LPA Framework

The core strength of our approach lies in the tripartite synergy of Loss, Prompt, and Augmentation (LPA). While current computer-aided methods [30,31] often prioritize visual feature depth at the expense of efficiency, and multimodal learning has succeeded in radiology [32,33], its application to polyp classification—particularly integrating imaging with structured clinical text—remains underexplored. LPA-Tuning CLIP addresses the “semantic void” inherent in unimodal systems.

The Medical-Aware Augmentation transcends generic data expansion by specifically simulating the physical properties of endoscopy, such as varying lighting angles and mucosal reflections. This ensures that the model remains robust against domain shifts that typically degrade the performance of models pre-trained on natural images. Furthermore, theStructured Clinical Semantic Template (SCST) provides a high-density information anchor. The three components of SCST (Entity, Quantification, and Context) are designed to function as an indivisible semantic unit rather than isolated modules. Just as a clinical diagnosis requires a subject, a description and a conclusion to be valid, the SCST relies on the interdependency of *E*, *Q*, and *C* to maintain logical integrity in the embedding space. By replacing ambiguous, short-text labels with WHO-compliant hierarchical prompts, we provide the vision encoder with explicit morphological cues (e.g., serrated architecture or villous structures). Finally, the CMPM with ID Loss functions as a precision alignment tool. While CMPM optimizes the global cross-modal probability distribution, the ID loss enforces strict intraclass compactness. This dual-loss strategy is pivotal for resolving the subtle inter-class similarities between adenomatous and hyperplastic polyps, which are often indistinguishable to general-purpose vision models.

### 5.2. Clinical Significance and Diagnostic Reliability

The clinical implications of this work are significant for the early detection and management of colorectal cancer. The accurate differentiation of polyp subtypes is not merely a classification task but a decisive factor in surgical planning and follow-up frequency. Adenomatous polyps, known for their malignant potential via the adenoma–carcinoma sequence, require mandatory excision, whereas hyperplastic polyps may only necessitate routine monitoring.

By achieving state-of-the-art performance, our framework serves as a reliable Computer-Aided Diagnosis (CAD) assistant that aligns with standard clinical workflows. The integration of structured pathological text ensures that the model’s “reasoning” is grounded in validated medical knowledge, thereby enhancing the interpretability of AI outputs for gastroenterologists. In practice, this could alleviate the cognitive load on endoscopists, reduce the miss rate of high-risk lesions during prolonged procedures, and provide a standardized “second opinion” that bridges the expertise gap between junior and senior physicians. Retrospective benchmarking against existing literature supports the model’s utility. Previous studies indicate that novice endoscopists often achieve an optical diagnosis accuracy ranging from 70% to 80% for diminutive polyps. With an accuracy of 85.8% and a recall (sensitivity) of 0.865, our LPA-CLIP framework outperforms the typical baseline of junior physicians. This performance gap suggests that the model has the potential to reduce the inter-observer variability inherent in visual diagnosis.

### 5.3. Limitations and Future Directions

Despite these promising results, several limitations warrant attention. First, the training and validation phases predominantly relied on single-center data. While external validation on large-scale public datasets (such as WCE or Kvasir) is desirable, these datasets currently lack detailed pathological text descriptions, which presents a challenge for direct transfer. The variability in endoscopic hardware (e.g., different imaging sensors from Olympus or Fujifilm) and bowel preparation quality necessitates further validation through multicenter trials to ensure universal applicability.

Second, the generation of SCST currently depends on manual expert annotation, which remains a labor-intensive bottleneck. Future research will explore the integration of Large Language Models (LLMs), such as GPT-4 or specialized medical LLMs, to automatically parse and structure raw pathology reports into high-quality templates. Additionally, we aim to extend the LPA-Tuning paradigm to real-time video-based polyp detection, incorporating temporal consistency and motion-blur robustness to support intraoperative decision-making.

Furthermore, regarding the transferability of our method, while the specific textual content of SCST is tailored to intestinal polyps, the underlying ’Entity–Quantification–Context’ (E⊕Q⊕C) structural logic is broadly applicable to other medical imaging tasks. This tripartite framework serves as a universal meta-template for pathological description: Entity (*E*) can be adapted to define lesion types in other domains (e.g., ’Glioblastoma’ in MRI). Histological Quantification (*Q*) can encode domain-specific grading criteria (e.g., ABCD rules for skin lesions). Clinical Contextualization (*C*) can specify prognosis or malignancy risk relevant to that disease.

Therefore, the SCST framework provides a transferable methodological paradigm that standardizes the injection of expert knowledge into vision-language models, extending beyond gastroenterology to broader diagnostic fields.

Finally, a direct prospective comparison with human endoscopists was not conducted in this study. Future work will prioritize a multi-center reader study to rigorously evaluate the model against physicians of varying experience levels.

## 6. Conclusions

In conclusion, LPA-Tuning CLIP establishes a new paradigm for polyp classification by integrating endoscopic visuals with structured pathology. Through its specialized augmentation, hierarchical prompt engineering, and label-aware loss functions, the model overcomes the limitations of traditional unimodal systems. Our findings demonstrate that cross-modal synergy, when grounded in clinical standards, provides a robust and interpretable pathway for advancing the precision of endoscopic diagnosis.

## Figures and Tables

**Table 1:** Number of samples and patients for each class in our dataset.

Class	Number of Samples	Number of Patients
Adenoma	154	39
Hyperplasia	147	36
Normal mucosa	88	16

**Table 2:** Comparison between standard class-name prompts and the proposed Structured Clinical Semantic Template (SCST) for each polyp category.

Category	Standard Prompt	Proposed SCST Prompt (E ⊕ Q ⊕ C)
Adenoma	A photo of an adenoma.	E: A photo of an adenoma. Q: Exhibiting tubular or villous architecture with low-grade intraepithelial neoplasia. C: Representing a common precancerous intestinal lesion.
Hyperplasia	A photo of hyperplasia.	E: A photo of a hyperplastic polyp. Q: Showing elongated crypts with a characteristic serrated or ’saw-tooth’ appearance. C: Identified as a generally benign mucosal proliferation.
Normal	A photo of normal mucosa.	E: A photo of normal intestinal mucosal tissue. Q: Characterized by regular crypt structures and smooth surface with no dysplasia. C: Indicating healthy intestinal mucosa without abnormalities.

**Table 3:** Accuracy results of the baseline models and the proposed model on the curated sets of the custom collected dataset.

Framework	Original		With Description		Ours (CMPM + ID + Desc.)	
	Acc.	F1-Score	Acc.	F1-Score	Acc.	F1-Score
ViT/B-16	76.3%	0.767	80.3%	0.804	82.3%	0.826
ViT/B-32	77.1%	0.773	81.2%	0.816	83.2%	0.836
ViT/L-14	78.6%	0.791	81.9%	0.821	85.8%	0.862
ViT/L-14@336px	75.7%	0.759	78.5%	0.788	81.2%	0.816

**Table 4:** Comparison with other advanced models.

Models	Accuracy	F1-Score	Recall
BiomedCLIP	83.9% ± 1.0%	0.843 ± 0.009	0.850 ± 0.013
MedCLIP	81.2% ± 1.1%	0.819 ± 0.012	0.823 ± 0.015
ConVIRT	78.5% ± 1.3%	0.792 ± 0.013	0.801 ± 0.015
PMC-CLIP	80.1% ± 0.9%	0.808 ± 0.011	0.816 ± 0.013
MedMamba	82.3% ± 1.2%	0.828 ± 0.013	0.832 ± 0.015
EfficientNet	75.1% ± 1.5%	0.761 ± 0.016	0.772 ± 0.017
Vision Transformer	76.8% ± 1.4%	0.773 ± 0.015	0.781 ± 0.016
ResNet	75.9% ± 1.6%	0.752 ± 0.018	0.755 ± 0.018
**Ours**	**85.8% ± 0.8%**	**0.862 ± 0.009**	**0.865 ± 0.011**

Note: Bold values indicate the best results.

**Table 5:** Ablation of the “Medical-Aware Augmentation”.

Models	Augmentation	Accuracy	F1-Score
LPA-Tuning CLIP	✔	**85.8% ± 0.8%**	**0.862 ± 0.009**
LPA-Tuning CLIP	✘	83.6% ± 1.0%	0.839 ± 0.013

Note: Bold values indicate the best results.

**Table 6:** Ablation of the “Medical Prompt”.

Models	Prompts	Accuracy	F1-Score
LPA-Tuning CLIP	SCST	**85.8% ± 0.8%**	**0.862 ± 0.009**
LPA-Tuning CLIP	Generic class	82.7% ± 1.0%	0.829 ± 0.011

Note: Bold values indicate the best results.

**Table 7:** Ablation of the “CMPM with ID Loss”.

Models	Loss	Accuracy	F1-Score
LPA-Tuning CLIP	CMPM with ID	**85.8% ± 0.8%**	**0.862 ± 0.009**
LPA-Tuning CLIP	InfoNCE	82.1% ± 1.2%	0.823 ± 0.012

Note: Bold values indicate the best results.

## Data Availability

The data that support the findings of this study are openly available in 12345Jun at https://huggingface.co/datasets/12345Jun/raw-data (accessed on 24 February 2026).
